# New tip-apex distance and calcar-referenced tip-apex distance cut-offs may be the best predictors for cut-out risk after intramedullary fixation of proximal femur fractures

**DOI:** 10.1038/s41598-021-04252-1

**Published:** 2022-01-10

**Authors:** Gaetano Caruso, Nicola Corradi, Antonio Caldaria, Daniele Bottin, Dario Lo Re, Vincenzo Lorusso, Chiara Morotti, Giorgia Valpiani, Leo Massari

**Affiliations:** 1 Department of Neurosciences and Rehabilitation, University of Ferrara, Via Aldo Moro 8, 44124 Ferrara, Italy; 2 Orthopaedic and Traumatology Unit, S. Anna University Hospital of Ferrara, Ferrara, Italy; 3 Research and Innovation Office, S. Anna University Hospital of Ferrara, Ferrara, Italy

**Keywords:** Trauma, Risk factors

## Abstract

Cut-out is one of the most common mechanical failures in the internal fixation of trochanteric hip fractures. The tip-apex distance (TAD), and the calcar-referenced tip apex distance (CalTAD) are the radiographic parameters that most predict the risk of cut-out. The optimal CalTAD value has not yet been defined, but the optimal TAD value is reported as 25 mm or less. However, this cut-off is highly specific but poorly sensitive. The aim of this study was to determine highly specific and sensitive TAD and CalTAD values and shed light on the role of other clinical variables. A total of 604 patients were included in this retrospective cross-sectional study. For each patient the following data were recorded: number of cut-out, AO/OTA classification, quality of the reduction, type of nail, cervicodiaphyseal angle, type of distal locking, post-operative weight-bearing, TAD and CalTAD values, and the position of the screw head in the femoral head according to the Cleveland system. The incidence of cut-out across the sample was 3.1%. The median TAD in the cut-out group was 38.72, while in the no cut-out group it was 22.16. The median CalTAD in the cut-out group was 39.34, while in the no cut-out group it was 22.19. The cut-off values for TAD and CalTAD with highest value of sensitivity and specificity for the risk of cut-out were 34.8 and 35.2, respectively. The incidence of cut-out can be reduced by performing careful minimal reduction and ensuring stable fixation by avoiding TAD > 34.8 mm and CalTAD > 35.2 mm.

## Introduction

Pertrochanteric fracture is common in elderly patients worldwide, and its incidence is expected to reach 6.3 million/year by 2050^1,2^. There is a close correlation between proximal femur fractures and increased risk of death and major complications, especially in elderly patients^3,4^.

Several authors have pointed out the benefits of surgery, and early surgical treatment to reduce related mortality is strongly recommended^5–7^. Nowadays, both extramedullary and intramedullary fixations are viable options^8^, but due to its biomechanical and biological advantages, intramedullary nailing has become the most used fixation device in pertrochanteric fractures worldwide, especially in unstable fractures^9,10^.

The most common cause of failure for this type of fixation is nail cut-out, which is defined as extrusion of the cephalic screw as a consequence of a varus collapse of the neck–shaft angle^11^. The prevalence of cut-out is estimated at between 1.85%–16.5%^12,13^, and several factors are thought to be related to this complication, including bone stock quality, cephalic screw positioning and length, tip–apex distance (TAD), calcar tip-apex distance (CalTAD) and fracture reduction^14–16^. In particular, Baumgaertner et al. identified a higher cut-out risk when the TAD was greater than 25 mm; the 25 mm TAD cut-off was confirmed by a biomechanical study by Kuzy et al., who introduced CalTAD as a new parameter in predicting cut-out. Consequently, most of current literature identifies a TAD shorter than 25 mm as correlating with a lower cut-out risk^15–17^, but while this cut-off is highly specific, it is poorly sensitive. Furthermore, several authors have reported that longer TADs and CalTADs do not, in fact, increase cut-out risk^18,19^.

Hence, the main aim of our study was to evaluate the best TAD and CalTAD cut-off values to define the cut-out risk. A further objective was to analyse the reliability of multiple other factors as predictors of the risk of lag-screw cut-out, specifically TAD and CalTAD values, Cleveland method, postoperative weight-bearing, type of fracture, quality of reduction, laterality and age.

## Materials and methods

This was a retrospective cross-sectional study on consecutive patients with pertrochanteric femur fracture treated with closed reduction and internal fixation with short intramedullary nails, admitted between January 2014 and December 2019 to our Orthopaedic and Traumatology Unit in the northeast of Italy (catchment area of roughly 350,000 inhabitants).

The adult patients undergoing surgery for pertrochanteric fractures were identified retrospectively from a hospital discharge database. The fractures were classified according to the AO-Müller/Orthopaedic Trauma Association fracture and dislocation classification (AO/OTA)^20^. Patients included in the study were those with an isolated proximal femur fracture type 31-A who underwent surgical intervention with proximal femur short nail (Gamma3 Trochanteric Nail 180 -Stryker – US or Trigen Intertan Intertrochanteric Antegrade Nail—Smith & Nephew—UK).

The *exclusion criteria* were as follows: proximal femoral fracture involving femur diaphysis or subtrochanteric fractures; pertrochanteric femoral fracture treated with open reduction and internal fixation with long intramedullary nails, extramedullary fixation or other devices; patients without minimum follow-up period of 3 months; pathological fractures induced by tumours or metastatic lesions; and poor-quality x-rays.

For each patient the following data were recorded: gender, ASA, operation time, hospitalization time, anaethesia, mortality, number of cut-outs or other complications; AO/OTA classification for trochanteric fractures (31-A1, 31-A2, 31-A3) based on pre-operative radiographic evaluation on anteroposterior and cross-table lateral projections; quality of the reduction (poor, acceptable, good) based on post-operative radiographic evaluation according to Baumgaertner’s criteria (normal or slight valgus alignment on antero-posterior radiograph, < 20° angulation on lateral radiograph and fracture gap equal to or less than 4 mm were considered as good reduction); type of nail (Gamma3 or Trigen Intertan), specifying the cervicodiaphyseal angle (120°, 125°, 130°) and the type of distal locking used (static, dynamic or unlocked); whether post-operative weight-bearing was allowed or not; TAD and CalTAD values; and the position of the screw head in the femoral head according to the Cleveland system^15,17,19,21^.

**Figure 1:**
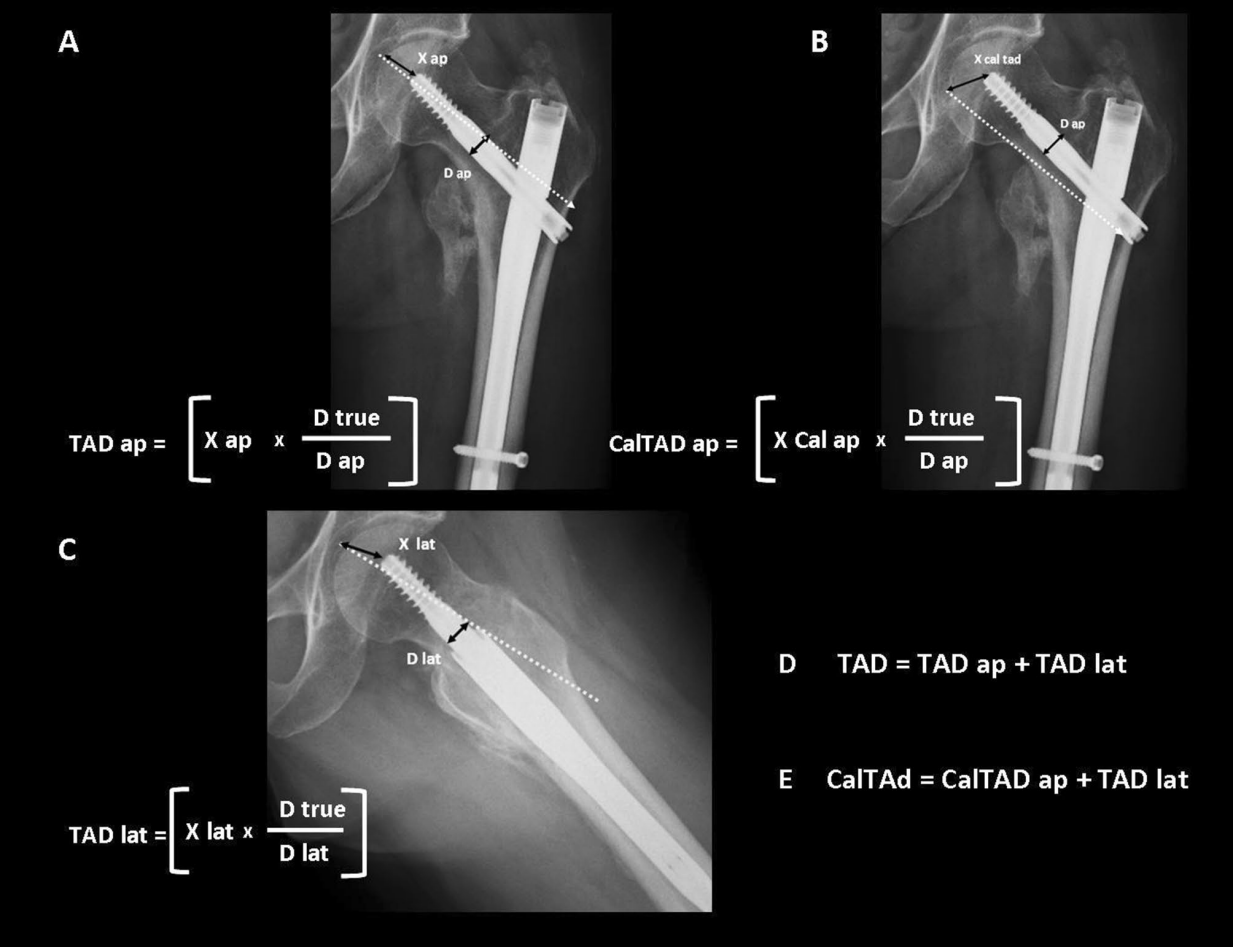
(**A**) Tip-apex distance calculated on anteroposterior radiograph (TAD ap); (**B**) Tip-apex distance as referenced to the calcar calculated on the anteroposterior radiograph (CalTAD ap); (**C**) Tip-apex distance calculated on the lateral radiograph (TAD lat); (**D**) Tip-apex distance (TAD); (**E**) Calcar-referenced tipapex distance (CalTAD). D true is the known diameter of the lag-screw (10.5 mm for Gamma3 nail, 15.5 mm for Trigen Intertan nail). D ap is the calculated diameter of the lag-screw on the anteroposterior radiograph. D lat is the calculated diameter of the lag-screw on the lateral radiograph.

TAD and CalTAD measurements were performed with the aid of Carestream Vue Picture Archiving and Communication system (PACs, version 12.2.5.00397) software. TAD and CalTAD were calculated on postoperative x-rays in two projections, namely a standard anteroposterior projection of the lower limbs rotated internally by 15°, and cross-table lateral projections with the contralateral limb flexed and abducted. A single observer (a consultant trauma surgeon) measured the TAD and the CalTAD, the screw position according to Cleveland method, and the fracture reduction, in order to eliminate inter-observer variability. Tad and CalTAD have been calculated with the formula shown in Fig. 1.

The study was approved by the local University-Hospital Human Subject Research Ethics Committee (Comitato Etico Indipendente di Area Vasta Emilia Centro—CE-AVEC 696/2020/Oss/AOUFe 23/07/2020), data collection and analysis were performed in compliance with the Declaration of Helsinki. The study was designed and written according to STROBE guidelines^22^.

### Statistical analysis

The Shapiro–Wilk test was used to test the normality of distribution of the continuous variables. In the presence of symmetrical distributions, the variables are represented as mean and standard deviation (SD), while non-normal distributions are expressed as a median and interquartile range (IQR). Categorical data are expressed as absolute numbers and percentages. Statistical comparisons of categorical variables were assessed using Pearson’s χ2 test or Fisher’s exact test, depending on the minimal expected count in each crosstab. Statistical comparisons of continuous variables were assessed using Student’s t -test for normally distributed variables, and the Mann–Whitney U-test in the case of non-normal distribution. Univariate analysis was used to estimate the ROC curves for TAD and CalTAD, in order to measure testing accuracy; the area under the curve (AUC) reflected test accuracy as follows: uninformative if AUC = 0.5; low accuracy if 0.5 < AUC ≤ 0.7; moderate accuracy if 0.7 < AUC ≤ 0.9; very high accuracy if 0.9 < AUC < 1; and perfect if AUC = 1. The thresholds for TAD and CalTAD were defined as the optimal cut-off that maximized the distance to the identity (diagonal) line in the ROC curve according to Youden’s J statistic. Two multivariate linear regression models were used to identify factors associated with the presence of cut-out, one using standard TAD and CalTAD thresholds, and the other one using the TAD and CalTAD thresholds determined in our analysis via Youden’s J statistic.

All analyses were performed using Stata 15.1 SE (Stata Corporation, College Station, Texas, USA); a *p* value < 0.05 was defined as statistically significant.

## Results

### Demographic results

**Figure 2:**
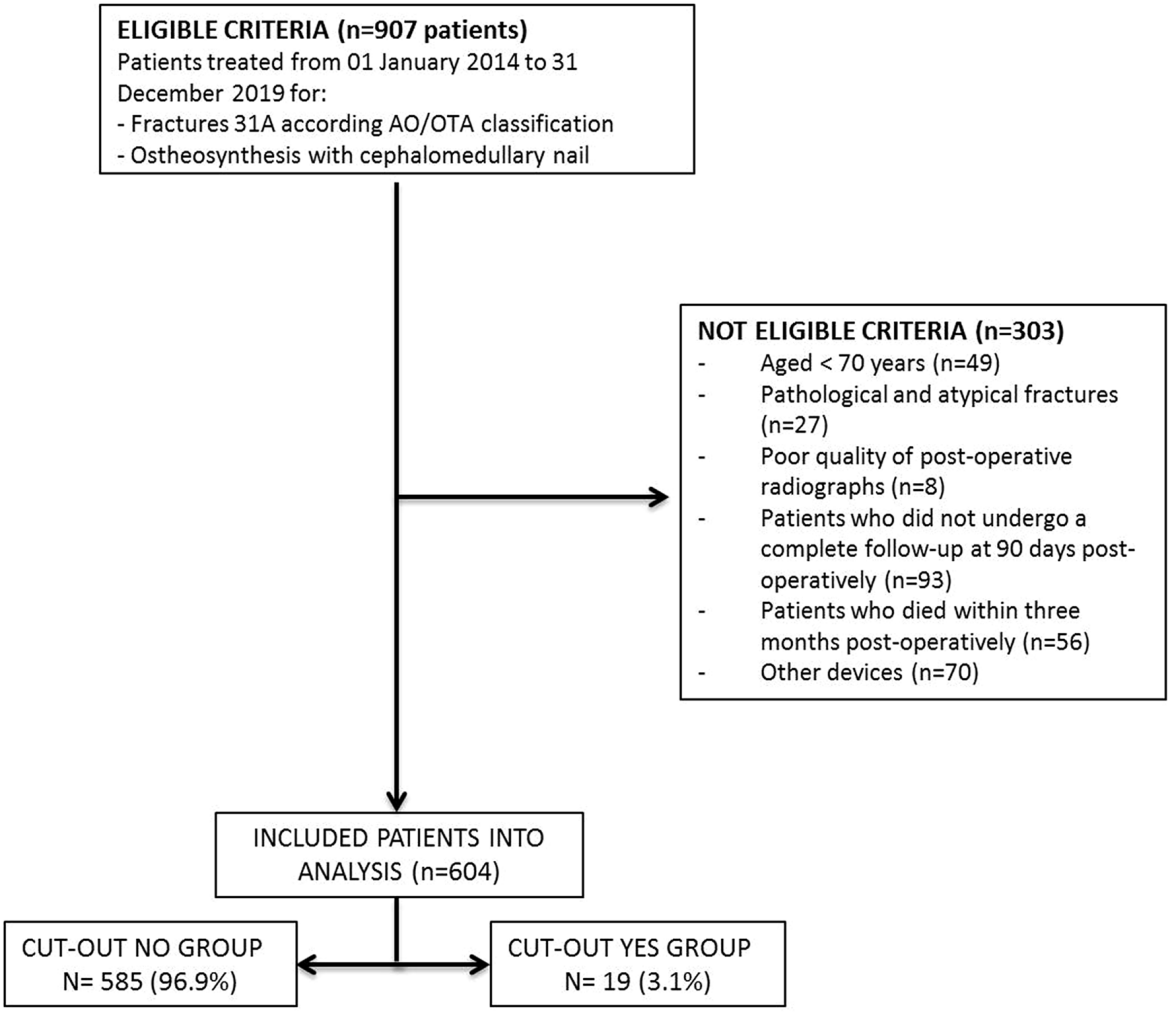
Flow chart describing the inclusion and exclusion criteria of the study.

A total of 907 patients were identified; of these, only 604 patients met the inclusion criteria and were therefore eligible for this study. Of 604 patients, 35% (N = 212) were males and 65% (N = 392) were females, with a mean age of 88 years. The average operative time was 37 min with a minimum of 20 min and a maximum of 90 min. 513 patients (85%) received subarachnoid anesthesia while 91 patients (15%) received general anesthesia. We recorded 138 patients (23%) with ASA 2, 383 patients (63%) with ASA 3 and 83 patients (16%) with ASA 4. The average time of hospitalization was 8 days. None of the 604 patients died in the first 3 months of follow-up while 59 patients (10%) died at 1-year follow-up. The most frequent causes of exclusion from the study were death, a follow-up period shorter than three months after surgical intervention, and the use of osteosynthesis devices different from those under study (Fig. 2). Among the 604 patients who were reviewed, lag-screw cut-out was observed in 19 cases—an incidence of 3.1%.

### Comparison of general factors associated with risk of cut-out

**Figure 3:**
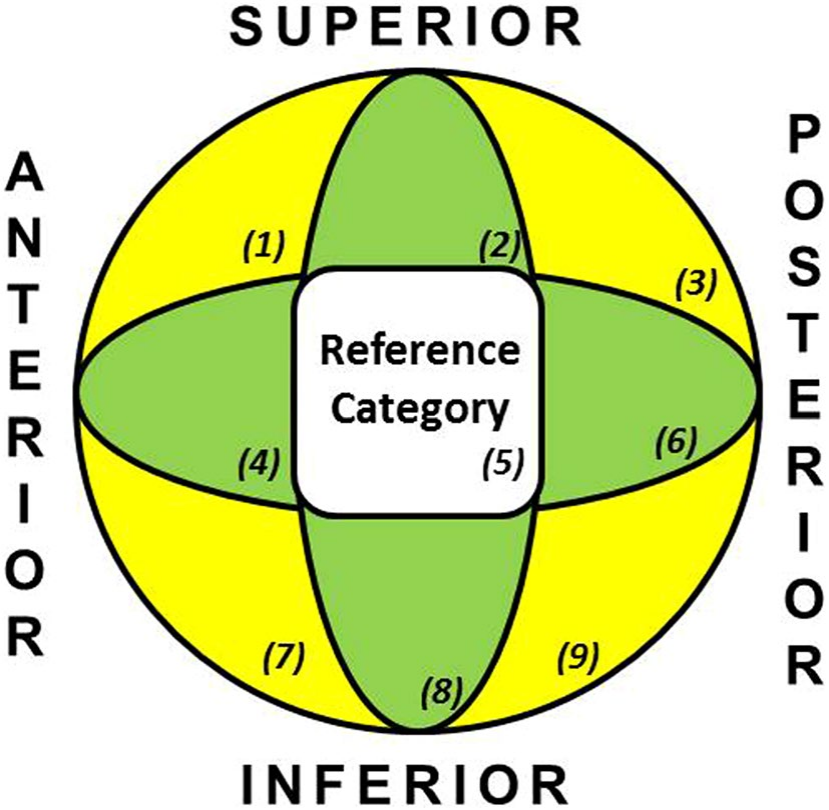
The modified Cleveland system used in our study. Nine areas we reduced to three, specifically the central (reference category) and two peripherals denoted “ + ” (in green) and “x” (in yellow).

Table 1 synthesizes the factors that we identified as possibly associated with the risk of cut-out. Specifically, while median age, fracture side and central column diaphyseal angle were not significantly associated with the risk of cut-out (*p* value > 0.05), the AO/OTA classification did display a statistically significant correlation with the cut-out risk in the A2 fracture type (*p* value < 0.05). Full post-operative weight bearing was also identified as a statistically significant risk factor for nail cut-out (*p* value 0.004). In addition, variables with an almost significant association with cut-out risk were the type of nail used (*p* value 0.058), static distal locking (*p* value 0.058) and poor-quality reduction (*p* value 0.061). The position of the screw according to the Cleveland system (Fig. 3) was strongly correlated with the cut-out risk (*p* value < 0.002), especially when screws were located in the periphery (*p* value < 0.023). Comparative analysis of the sample divided on the basis of the presence or absence of cut-out demonstrated a significant difference related to both TAD and CalTAD.

**Table 1:** demographic data and baseline characteristics of all patients with trochanteric fractures.

	CUT-OUT NO (n = 585)	CUT-OUT YES (n = 19)	TOTAL (n = 604)	*p* value
Age, mean (IQR, Q1to Q3)	88 [82 93]	89 [83 95]	88 [82 93]	0.4658
**Side (n, %)**				0.737
right	285 (48.7)	10 (52.6)	295 (48.8)	
left	300 (51.3)	9 (47.4)	309 (51.2)	
**AO/OTA Classification (n, %)**				
A1	334 (57.1)	7 (36.8)	341 (56.5)	0.08
A2	228 (39)	12 (63.2)	240 (39.7)	0.034
A3	23 (3.9)	0 (0)	23 (3.8)	0.999
**Intramedullary Device (n, %)**				0.058
Gamma 3	302 (51.6)	14 (73.7)	316 (52.3)	
Trigen Intertan	283 (48.4)	5 (26.3)	288 (47.7)	
**Distal Locking (n, %)**				
Static	491 (83.9)	15 (78.9)	506 (83.8)	0.058
Dynamic	92 (15.7)	4 (21.1)	96 (15.9)	0.523
Unlocked	2 (0.4)	0 (0)	2 (0.3)	0.999
**Centre-column-diaphyseal (CCD) angles (n, %)**				
120°	63 (10.8)	1 (5.3)	64 (10.6)	0.709
125°	477 (81.7)	17 (89.5)	494 (81.9)	0.550
130°	44 (7.5)	1 (5.3)	45 (7.5)	0.712
**Other complications (n, %)**				0.431
Yes	569 (97.6)	18 (94.7)	587 (97.5)	
No	14 (2.4)	1 (5.3)	15 (2.5)	
**Modified Cleveland Sistem (MSC) (n, %)**				
5	299 (51.1)	3 (15.8)	302 (50)	0.002
2–4-6–8	195 (33.3)	9 (47.4)	204 (33.8)	0.222
1–3-7–9	91 (15.6)	7 (36.8)	98 (16.2)	0.023
**Quality of reduction (n, %)**				0.061
good	370 (63.2)	8 (42.1)	378 (62.6)	
acceptable, poor	215 (36.8)	11 (57.9)	226 (37.4)	
**Post-operative weight-bearing (n, %)**				0.004
yes	469 (80.2)	10 (52.6)	479 (79.3)	
no	116 (19.8)	9 (47.4)	125 (20.7)	
TAD median (IQR, Q1 to Q3) (mm)	22.16 [16.66 28.6]	38.72 [30.59 46.23]	22.58 [17 28.97]	< 0.001
CalTAD median (IQR, Q1to Q3) (mm)	26.19 [21.77 31.19]	39.34 [36.76 46.87]	26.42 [21.93 31.5]	< 0.001

IQR, interquartile range; AO/OTA Classification, AO Foundation and Orthopaedic Trauma Association classification system; TAD, tip-apex distance; CalTAD, calcar-referenced tip-apex distance.

### Comparison between TAD and CalTAD

**Figure 4:**
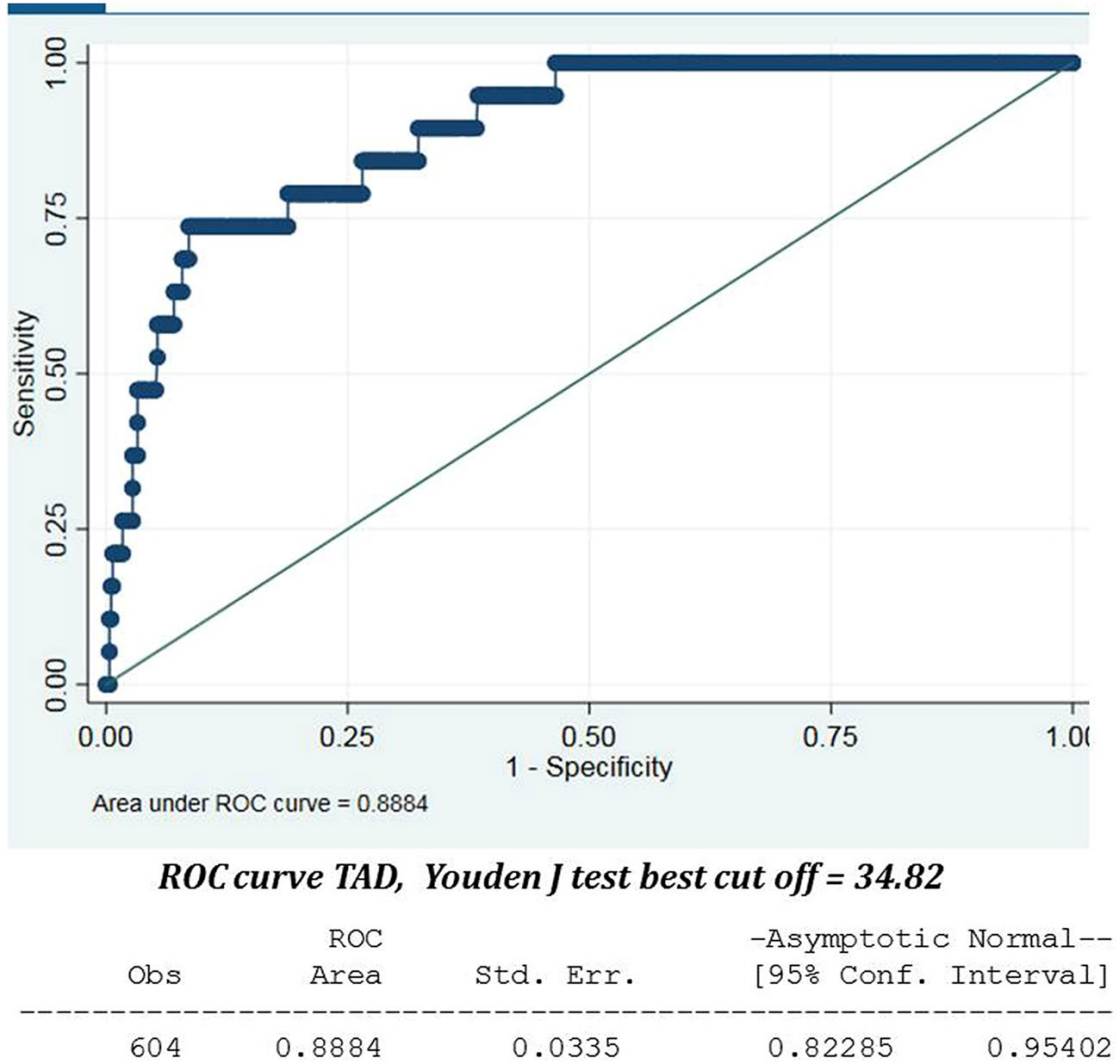
ROC curve TAD. The Youden’s test shows that the more sensitive and specific value of TAD for predict the risk of cut-out is 34.8.

**Figure 5:**
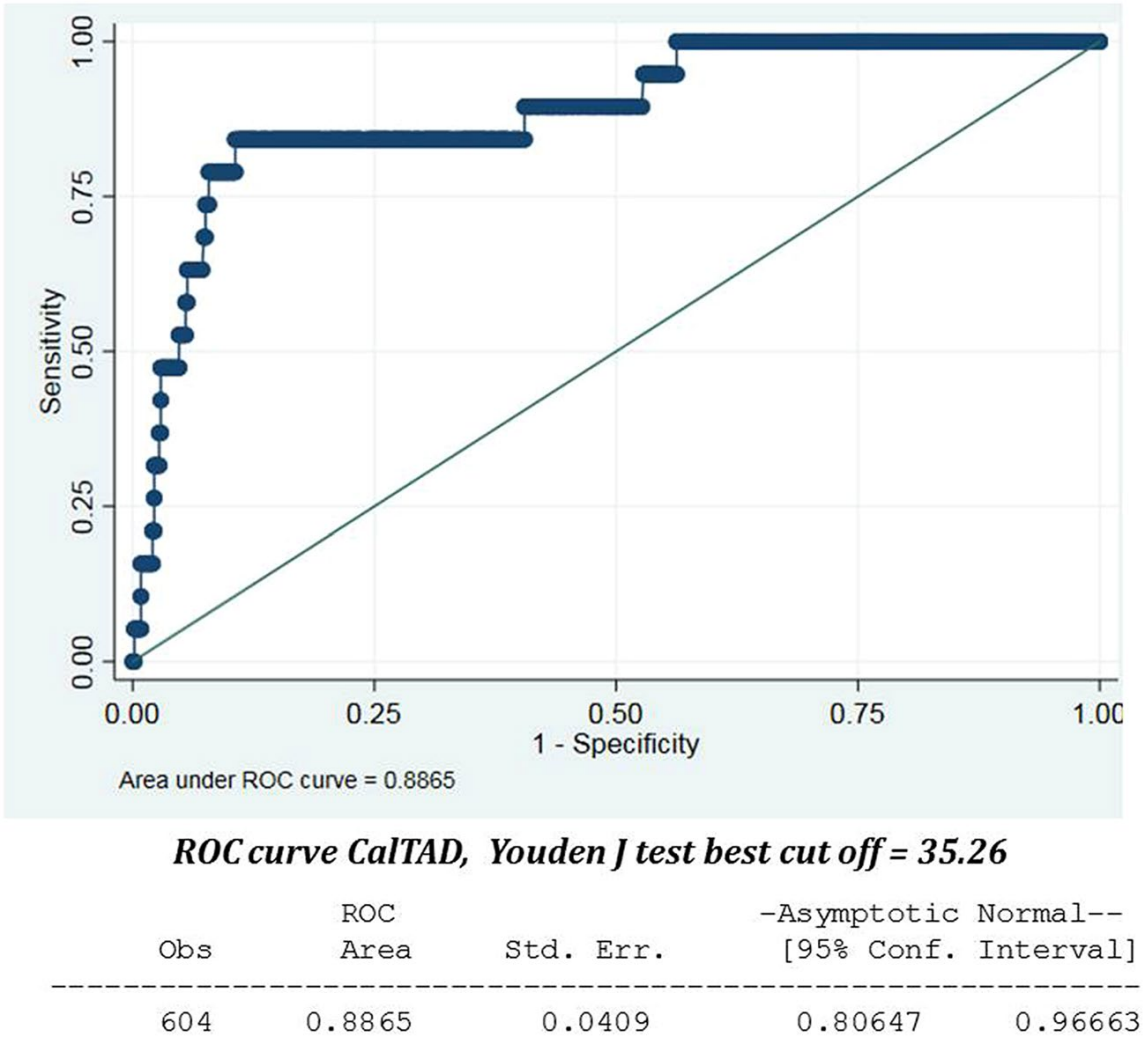
ROC curve CalTAD. The Youden’s test shows that the more sensitive and specific value of CalTAD for predict the risk of cut-out is 35.2.

Although the median overall TAD was 26.42 mm, the median TAD in the cut-out group was 38.72 mm (Q1 to Q3 30.59–46.23), while in the no cut-out group it was 22.16 mm (Q1 to Q3 16.66–28.6) (*p* value < 0.001). Similarly, while the median overall CalTAD was 26.42 mm, the median CalTAD in the cut-out group was 39.34 mm (Q1 to Q3 36.76–46.87), while in the no cut-out group it was 22.19 mm (Q1 to Q3 21.77–31.19) (*p* value < 0.001). The univariate logistic regression model showed that TAD of 25 mm implied 13.8% cut-out risk while TAD of 34.8 mm implied 29.3% cut-out risk. CalTAD of 35.2 mm connoted 42.7% cut-out risk while for CalTAD of 25 mm the risk could not be calculated (Table 2). The multivariate logistic regression model, considering TAD > 25 mm and CalTAD of 25 mm (Table 3), yielded an OR of 3.54 for the TAD, with a* p* value which was not statistically significant, while the OR for the CalTAD could not be calculated (Table 4). The application of the Youden test to detect the highest value of sensitivity and specificity showed that the best cut-off values are 34.8 mm for TAD (Fig. 4), and 35.2 mm for CalTAD (Fig. 5). In fact, the multivariate logistic regression model considering TAD > 34.8 mm and CalTAD > 35.2 mm yielded an OR of 4.40 (*p* value 0.032) for the former and an OR of 17.76 (*p* value < 0.001) for the latter. None of the other factors analysed for possible correlation with cut-out risk yielded a statistically significant result upon multivariate analysis (*p* value > 0.05).

**Table 2:** The univariate analysis, considering TAD > 25 mm, CalTAD > 25 mm, TAD > 34.8 mm and CalTAD > 35.2 mm.

Variable	OR (95% CI)	*p* value
TAD 25 mm	13.8 (3.18–60.73)	< 0.001
TAD 34.8 mm	29.32 (10.15–84.68)	< 0.001
CalTAD 25 mm	–	–
CalTAD 35.2 mm	42.67 (12.11–150.39)	< 0.001

**Table 3:** The multivariate logistic regression model, considering TAD > 25 mm and CalTAD > 25 mm.

		Cut-off TAD 25 and Cut-off CalTAD 25			
		Odds Ratio (OR)	95% confidence interval (CI)		*p* value
			Lower	Upper	
TAD	Ref (< 25 mm)	3.54	0.77	16.31	0.105
CalTAD	Ref (< 25 mm)	–	–	–	–
Age		1.01	0.96	1.06	0.105
AO/OTA Classification	Ref (A1 + A3)	1.89	0.66	5.45	0.237
Intramedullary Device	Ref (Gamma3)	0.76	0.44	1.34	0.348
MSC					
	2–4-6–8	3.15	0.81	12.21	0.097
	1–3-7–9	3.79	0.91	15.77	0.067
Quality of reduction	Ref (good)	1.37	0.48	3.92	0.533
Weight Bearing	Ref (no)	0.55	0.19	1.58	0.266

**Table 4:** The multivariate logistic regression model, considering TAD > 34.8 mm and CalTAD > 35.2 mm.

Variable		Cut-off TAD 34.8 and Cut-off CalTAD 35.2			
		Odds Ratio (OR)	95% confidence interval (CI)		*p* value
			Lower	Upper	
TAD	Ref (< 34.8 mm)	4.40	1.13	17.03	0.032
CalTAD	Ref (< 35.2 mm)	17.76	3.83	82.22	< 0.001
Age		1.01	0.96	1.07	0.696
AO/OTA Classification	Ref (A1 + A3)	2.77	0.84	9.13	0.093
Intramedullary Device	Ref (Gamma3)	0.71	0.39	1.29	0.256
MSC					
	2-4-6-8	1.79	0.39	8.22	0.450
	1-3-7-9	2.15	0.41	11.22	0.362
Quality of reduction	Ref (good)	1.6	0.49	5.14	0.429
Weight Bearing	Ref (no)	0.75	0.21	2.64	0.658

## Discussion

Cut-out is one of the complications of cephalomedullary nailing of the proximal femur fracture most feared by surgeons due to its great impact on functional recovery and life expectancy in elderly patients^11,23^. Although this study is limited by the systematic bias associated with retrospective studies, and the fact that all statistical data are based on the small number of cut-outs in our case series, it revealed several interesting findings. In 1995, Baumgaertner et al. introduced the definition of the tip–apex-distance (TAD) as the sum of the distance, measured on anteroposterior and lateral radiographs, between the tip of the lag screw and the femur head apex. They defined the optimal cut-off for TAD as 25 mm^15^. In 2012, Kuzyk et al. identified a new parameter associated with lag-screw cut-out risk, which they termed the calcar tip–apex distance (CalTAD), defined as the distance between the lag screw tip and the calcar femorale^16^. However, no definitive CalTAD cut-off has yet been established^13,16^, and CalTAD does not appear to be superior than TAD in predicting cut-out^24,25^.

Several authors have highlighted that Baumgaertner’s TAD cut-off of 25 mm is not supported by clinical evidence. Yam et al. raised the traditional TAD cut-off from 25 to 27 mm^18^, and others have reported that a limit of 25 mm has no biomechanical justification^18,26,27^.

In our analysis, TAD > 25 mm yielded an OR of 3.54 with a p-value that is not statistically significant, and an unmeasurable OR for CalTAD < 25 mm, as no cut-out was seen with CalTAD < 25 mm. In contrast, TAD > 34.8 mm yielded an OR of 4.40 (p-value 0.032) and CalTAD > 35.2 mm an OR of 17.76 (*p* value < 0.001). Therefore, limited to our study, there are reasonable grounds for raising the TAD cut-off from 25 mm to 34.8 mm, and an acceptable CalTAD cut-off would appear to be 35.2 mm. Indeed, these new cut-offs intercepted most of the lag-screw cut-outs encountered in our study. Specifically, our cohort presented 5 cut-outs with TAD lower than 34.8 mm and 14 cut-outs with TAD greater than 34.8 mm, while 3 cut-outs were reported with CalTAD < 35.2 mm and 16 with CalTAD > 35.2 mm.

Our results show that factors such as age, laterality and neck-shaft angle do not appear to promote cephalic-screw cut-out. However, the association of post-operative weight-bearing with the risk of cut-out was found to be statistically significant, with a higher cut-out rate in the group in which bearing was not granted immediately after surgical intervention than in the group allowed immediate full post-operative weight bearing. This can be explained by the fact that bearing was allowed on base of the subjective “sensation” of stability that the surgeon perceived during the surgery, together with the quality of the reduction assessed on post-operative x-rays. In other words, patients whose fractures displayed poor reduction and a feeling of poor bone tightness, two variables that have historically been considered predictive of cut-out, would be advised not to bear weight immediately post-operatively.

Indeed, in our sample the quality of reduction, along with distal locking and the choice of nail, displayed a correlation with the risk of cut-out approaching statistical significance. Specifically, we found a higher percentage of cut-out associated with poor or acceptable reductions, and with dynamic distal locking and the use of a mono-cephalic nail. None of these variables displayed a significant statistical correlation with cut-out in the multivariate statistical analysis. Similar results were also found in other recent studies, suggesting that these variables are still able to increase the risk of cut-out, but only if associated with stronger predictive factors^14,28–30^.

On this topic, it is widely reported in the literature that positioning the lag screw in the upper quadrants, according to the Cleveland system diagram^12,14–19,21^, increases the risk of cut-out. However, it is still under discussion whether the best screw position is the centre-centre or the inferior-centre quadrant. In our analysis, position 5 demonstrated a statistically significant correlation with a reduction in the risk of cut-out (*p* < 0.002). In contrast, positioning the lag screw in peripheral positions (zones 1, 3, 7 or 9) displayed a statistically significant correlation with an increased risk of cut-out (*p* < 0.02). These findings mirror those reported in 2017 by Caruso et al. on a series of 571 patients^19^.

These findings have an important impact on clinical practice. Aihara et al. suggested the importance of advocating weight-bearing restrictions in those patients at high risk of cut-out^31^. Pending validation and further studies, the new TAD and CalTAD cut-offs we propose would seem to be a safer and more selective method for the establishment of weight-bearing and mobilization restrictions, allowing these restrictions to be imposed on a smaller patient cohort. In fact, in our cohort TAD > 34.8 mm would suggest restrictions for only 65 patients instead of the 239 presenting TAD > 25 mm, while a CalTAD > 35.2 mm cut-off would have imposed weight-bearing restrictions on only 81 patients rather than the 350 patients that displayed CalTAD > 25 mm.

### Limitations

This study is limited by the systematic bias associated with retrospective studies, and the fact that all statistical data are based on the small number of cut-outs in our case series, it revealed several interesting findings. Furthermore, human error in performing TAD and CalTAD radiographic measurements could have represented sources of bias.

## Conclusions

In conclusion, limited to the results of our study, in order to reduce the incidence of cut-out, particularly in 31-A2 fractures, it is advisable to perform careful, minimal reduction, and achieve stable synthesis, avoiding TAD > 34.8 mm and CalTAD > 35.2 mm. Moreover, if possible, it appears to be preferable to use a nail with double cephalic screw and static distal locking, and to position the screw in Cleveland zone 5. In the event of poor reduction and/or unstable synthesis, unconstrained weight bearing alone is not sufficient to prevent the onset of cut-out. Limited to our study, the value of CalTAD seems to be more effective in predicting the risk of cut-out in the postoperative period than the TAD value. However, the differences between the two results are minimal and limited to our case series. In addition, even in the literature there are no studies demonstrating a greater sensitivity and specificity of CalTad than TAD and vice versa.

### Ethics approval

The study was approved by the local Ethics Committee (Comitato Etico Indipendente di Area Vasta Emilia Centro—CE AVEC: 696/2020/Oss/AOUFe 23/07/2020).

### Consent to participate

Data collection and analysis was performed in compliance with the Declaration of Helsinki.

### Informed consent

Informed consent to participate was obtained for as many patients enrolled in the retrospective study as possible. They are available upon request.

## Data Availability

The datasets used and/or analysed during the current study are available from the corresponding author on reasonable request.
